# Toll-Like Receptors and Relevant Emerging Therapeutics with Reference to Delivery Methods

**DOI:** 10.3390/pharmaceutics11090441

**Published:** 2019-09-01

**Authors:** Nasir Javaid, Farzana Yasmeen, Sangdun Choi

**Affiliations:** Department of Molecular Science and Technology, Ajou University, Suwon 16499, Korea

**Keywords:** Toll-like receptor, immunological disease, therapeutic, drug delivery method

## Abstract

The built-in innate immunity in the human body combats various diseases and their causative agents. One of the components of this system is Toll-like receptors (TLRs), which recognize structurally conserved molecules derived from microbes and/or endogenous molecules. Nonetheless, under certain conditions, these TLRs become hypofunctional or hyperfunctional, thus leading to a disease-like condition because their normal activity is compromised. In this regard, various small-molecule drugs and recombinant therapeutic proteins have been developed to treat the relevant diseases, such as rheumatoid arthritis, psoriatic arthritis, Crohn’s disease, systemic lupus erythematosus, and allergy. Some drugs for these diseases have been clinically approved; however, their efficacy can be enhanced by conventional or targeted drug delivery systems. Certain delivery vehicles such as liposomes, hydrogels, nanoparticles, dendrimers, or cyclodextrins can be employed to enhance the targeted drug delivery. This review summarizes the TLR signaling pathway, associated diseases and their treatments, and the ways to efficiently deliver the drugs to a target site.

## 1. Introduction

The immune system in an organism is the protective system combating pathogenic and/or abnormal conditions. Innate and adaptive immune systems are two basic components in vertebrates. Adaptive immunity is mediated by T and B lymphocytes with the help of their antigen-specific receptors that are encoded as a result of hypermutability and rearrangements in a genomic region of the organism. Nevertheless, evolutionarily conserved innate immunity is considered the first line of defense against invading pathogens [1]. For a long time, innate immunity has been regarded as a nonspecific response accomplished by phagocytes such as neutrophils and macrophages. On the other hand, pioneering studies on the loss-of-function mutations in *Drosophila melanogaster* and mice highlighted the molecular mechanisms for recognition of the pathogens and activation of the immune response [2,3,4]. Innate-immunity cells such as dendritic cells (DCs), macrophages, and neutrophils respond to invading pathogens by recognizing their associated markers, known as pathogen-associated molecular patterns (PAMPs). These PAMPs are specifically recognized by relevant cognate receptors known as pattern recognition receptors (PRRs). There are two main categories of theses PRRs: membrane-bound and cytoplasmic. The membrane-bound PRRs include Toll-like receptors (TLRs) and C-type lectin receptors. Cytoplasmic PRRs include NOD-like receptors and RIG-I-like receptors.

The very first human biological therapeutic obtained from gene manipulation was human insulin (Humulin^®^) generated by Eli Lilly at Genentech and approved in 1982 by the US Food and Drug Administration (FDA) [5]. The application of peptides as therapeutic agents has gradually gained popularity and expanded with innovation in drug improvement and treatment archetypes [6]. There has been growing interest in the development of targeted therapeutic drugs in the last three to four decades, which encouraged the progress in monoclonal antibodies, especially for the treatment of cancer and immunological diseases [7]. Nowadays, fruitful results are obtained in clinical trials on different diseases including Parkinson’s disease [8], Leber’s amaurosis [9], hemophilia B [10], thalassemia [11], hereditary immunodeficiency diseases [12,13,14], leukodystrophy [15], B-cell cancers, and heart failure [16]. For those drugs that require periodic or difficult delivery (such as ocular injectable drugs) and have poor pharmacodynamics, there is a precise solution: to construct molecules with high in vivo stability and potentially low immunogenicity. Some frequently used methods of molecular half-life expansion include the addition of stabilizing peptides, creation of Fc fusion proteins, and the inclusion of biomolecules into many discrete nanoparticle systems [17]. In a few medical conditions, where drugs do not pass certain barriers (e.g., the blood–cerebrospinal fluid barrier or blood–brain barrier) or do not show binding or affinity to a definite target molecule, the ligand-modified type of nanocarriers has been used to allow a drug to cross the cell membrane and to enable organized drug delivery in a specific state. For instance, hyaluronic acid (a polysaccharide from the extracellular matrix) has been used as an appended ligand in various nanocarriers, thereby yielding good outcomes, e.g., enhancing antitumor activity against breast cancer cells [18] and melanoma stem-like cells [19], in addition to lowering the immunogenicity of the formed protein corona [20], promotion of intravitreal drug distribution for retinal gene therapy [21], and targeting of pulmonary adenocarcinoma cells [22]. Biological therapeutics can be generally classified into three big groups based on their physical properties and mode of action. The first group includes peptides and small proteins such as cytokines, growth factors, and hormones. The second group includes therapeutic proteins which are nonimmunogenic such as blood factors, therapeutic replacement enzymes, and anticoagulants. The third group contains the most rapidly growing class of biotherapeutic drugs: therapeutic antibodies and Fc-like fusion proteins [23]. Hundreds of monoclonal antibodies and fusion proteins are in the process of clinical evaluation [24].

Biologics are currently the rapidly developing group of pharmaceuticals for the treatment of a number of chronic and deadly diseases. They consist of a varied group of biological substances that largely include proteins, nucleic acids, viral particles, whole cells, and vaccines [25]. Many biologics have made their way to the market; they mainly include blood factors, antibody-based drugs, anticoagulants, engineered protein scaffolds, bone morphogenetic proteins (BMPs), enzymes, Fc (crystallizable fragment of an antibody), hormones, growth factors, fusion proteins, interferons, thrombolytics, and interleukins [5]. The biological therapeutics’ production and development encounter various challenges that are quite different from those faced by classical small-molecule drugs [26]. In general, biologics are designer drugs whose mode of action in an underlying disease pathophysiology is usually better understood than that of small-molecule drugs [27]. The benefits of biologics are associated with the considerable technology and tool evolution for their development over the past three decades. Various protein engineering platforms, diverse selection technologies, new production systems, a profusion of biotherapeutics’ formats and scaffolds, and new methods for increasing aggregation resistance and stability have resulted in a new era of therapeutic candidates [23]. The biological therapeutics have burst onto the scene undoubtedly because of a greater chance of being first-class therapeutics in comparison with small-molecule drugs, considering their quality and novelty [27]. Despite their good properties, they are underestimated in the pharmaceutical market. The reason might mainly be their poor bioavailability, which necessitates the use of some special medical devices (e.g., inhalers) and nonoral administration. Another drawback is their lower metabolic stability because of proteolytic degradation. In addition, the cost to synthesize some biopharmaceutics is high. Their pharmacokinetics can be improved by conjugating them with other substances or by transforming them into small molecules [28].

Here, we review TLRs and their signaling pathways, their involvement in various diseases, and relevant reported therapeutic drugs, including biologics, small-molecule inhibitors, and nucleic acids. Moreover, we explain various drug delivery approaches that could be used to enhance the efficacy of TLR-targeting drugs.

## 2. Toll-Like Receptors and Their Signaling Pathways

**Figure 1:**
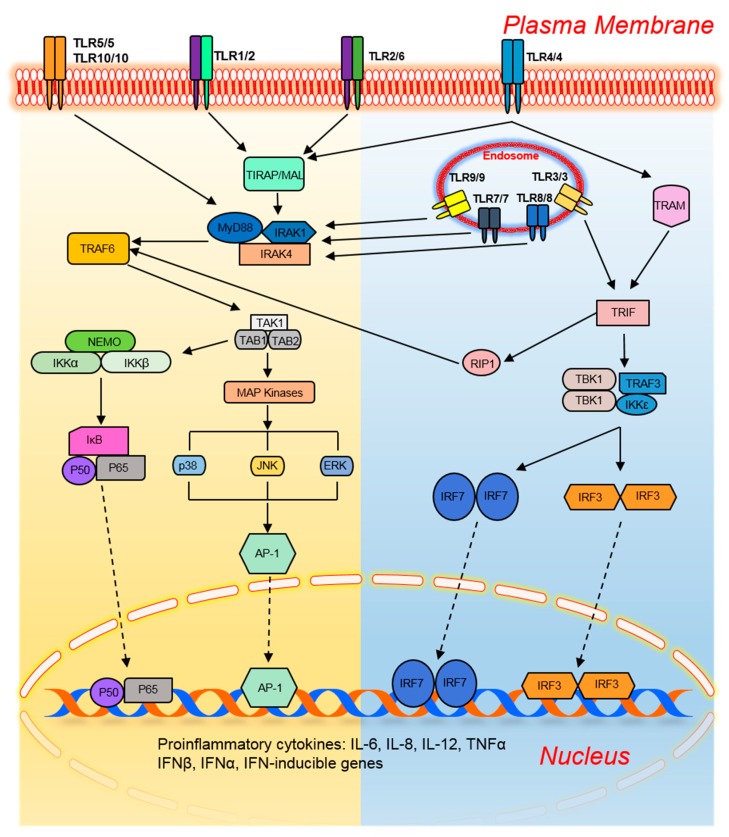
A generalized Toll-like receptor signaling mechanism, adapted from [30]. TLRs are subdivided into two main categories based on their location, cell surface or endosomal; they are activated by their respective PAMPs and damage-associated molecular patterns. After recognizing respective ligand(s) via extracellular domains, TLRs form homo- or heterodimers and interact with cytoplasmic adaptor molecules (myeloid differentiation primary response protein 88 (MyD88), TIRAP (MAL), TRIF, or TRAM) through their cytoplasmic TIR domain. Most of TLRs activate downstream signaling in a MyD88-dependent manner with or without recruiting MAL; however, TLR3 (only) and TLR4 (alternatively) use TRIF/TRAM adaptors to activate IRFs. The activation of a TLR signaling pathway leads to the translocation of transcription factors (NF-κB (p50 or p65), AP-1, IRF3, and IRF7) into the nucleus and allows them to bind the target sequence(s) of inflammatory and interferon-responsive genes. Abbreviations: TLR, Toll-like receptor; TIR, Toll/interleukin-1 receptor domain; MAL, MyD88 adaptor like; MyD88, myeloid differentiation primary response 88; TRAM, TRIF-related adaptor molecule; TRIF, TIR domain-containing adaptor inducing interferon β; IRAK, interleukin receptor-associated kinase; RIP, receptor-interacting protein; TRAF, tumor necrosis factor receptor (TNFR)-associated factor; TAB, TAK-1–binding protein; TAK1, transforming growth factor β-activated kinase 1; TBK1, TANK-binding kinase 1; NEMO, NF-κB essential modulator; IκB, inhibitor of κB; IKK, inhibitor of κB kinase; MAP, mitogen-activated protein; ERK, extracellular signal–regulated kinase; JNK, c-Jun N-terminal kinase; p38, protein 38; AP-1, activated protein 1; IFN, interferon; IL, interleukin; IRF, interferon response factor; NF-κB, nuclear factor κB; TNF-α, tumor necrosis factor α.

To date, 10 members of the TLR family in humans and 13 in mice have been identified. They are present on different immune cells and recognize their respective ligands (Table 1). TLRs trigger specific intracellular signaling pathways after recognition of microbial pathogens, resulting in the release of inflammatory cytokines, chemokines, and type I interferon (IFN). Moreover, TLRs link the innate immune response and adaptive immune response by upregulating the costimulatory molecules on antigen-presenting cells (DCs), a phenomenon known as DC maturation [29]. Activation of common signaling pathways by TLRs results in the production of various cytokines including tumor necrosis factor alpha (TNF-α), IL-1β, IL-6, and IL-12 as well as response elements from alternate pathways that prevent a microbial attack (Figure 1) [30]. Specifically, a type I IFN (IFNα and IFNβ)-based antiviral response is provoked by the activation of TLR3, TLR4, TLR7, TLR8, and TLR9 (Figure 1).

The extracellular leucine-rich repeats of TLRs recognize the pathogens while the transmembrane and cytoplasmic Toll/interleukin-1 receptor (TIR) domains initiate intracellular signaling [30]. All the TLRs provoke an inflammatory and protective response by activating nuclear factor-κB (NF-κB), activating protein-1 (AP-1), interferon regulatory factor (IRF) 3, and IRF7. The dimeric transcription factor NF-κB belongs to the Rel domain-containing family, which includes RelB, c-Rel, p65/RelA, p50/NF-κB1, and p52/NF-κB2 [31]. NF-κB is a heterodimer composed of subunits p50 and p65 and is sequestered in an inactive form by inhibitor of NF-κB (IκB) in unstimulated cells.

Upon TLR-mediated stimulation, phosphorylation of IκB is performed at serine residues by the IKK complex (IKKα, IKKβ, and IKKγ/NEMO) which targets IκB for ubiquitination-based degradation by 26S proteasome. This change frees NF-κB to relocate into the nucleus and bind to inflammation-responsive genes. On the other hand, dimeric basic leucine zipper (bZIP) protein AP-1 is composed of the members of Fos, Jun, Maf, and activating transcription factor (ATF) subfamilies, which bind to a cAMP response element and TPA-response element [32]. Among them, c-Jun plays a central role in the initiation of an inflammatory response. TLR-associated AP-1 activation is mostly mediated by MAP kinases including p38, c-Jun N-terminal kinase (JNK), and extracellular signal–regulated kinase (ERK). The stimulation of cells with lipopolysaccharide (LPS) or poly-IC and/or a virus attack activates IRF3 and IRF7, which control the expression of type I interferon (IFN). The structurally related proteins IRF3 and IRF7 are present in the cytoplasm of unstimulated cells and relocate into the nucleus after stimulation-mediated phosphorylation by TANK-binding kinase (TBK)1, noncanonical IKKs, and IKKi and activate the expression of target genes [33,34]. The TLR-mediated activation of these transcription factors is divided into two main categories: MyD88 dependent and TRIF dependent.

### 2.1. The MyD88-Dependent Pathway

The preliminary activation of intracellular TLR signaling is implemented by heterophilic interaction among TIR domains of TLRs and cytoplasmic adaptor proteins such as MyD88, TIR domain-containing adaptor protein (TIRAP/Mal), TIR domain-containing adaptor inducing IFNβ (TRIF/TICAM1), and TRIF-related adaptor molecule (TRAM/TICAM2; Figure 1) [30]. MyD88 is considered a central adaptor shared by all TLRs except TLR3. The interaction of TLRs and MyD88 recruits interleukin 1 receptor-associated kinase (IRAK) family members: IRAK1, IRAK2, IRAK4, and/or IRAK-M. IRAK1 and IRAK4 positively regulate TLR signaling through their intrinsic serine/threonine kinase activities unlike IRAK2 and IRAK-M, which perform negative regulation because of a lack of this kinase activity [35,36]. TLR stimulation dissociates IRAK1 and IRAK4 from MyD88 after their phosphorylation, which in turn activates tumor necrosis factor receptor-associated factor 6 (TRAF6). TRAF6 contains a conserved E3 ubiquitin ligase N-terminal RING domain, which allows it to form a complex with Ubc13 and Uev1A with subsequent synthesis of lysine63-linked polyubiquitin chains [37]. TRAF6 ubiquitination activates a MAP kinase kinase kinase (MAP3K) family member, transforming growth factor β–activated protein kinase (TAK) 1, which forms a complex with TAB-1, -2, and -3 [37]. In particular, zinc-finger domains of TAB2 and TAB3 allow them to interact with lysine63-linked polyubiquitin chains; this event activates TAK1 [37]. TAK1 activation causes the IKK complex to eventually activate NF-κB. Simultaneously, TAK1 phosphorylates MKK3 and MKK3, members of the MAP kinase kinase family, for subsequent activation of JNK and p38. ERK is stimulated through the TLR-mediated activation of MEK1 and MEK2. These mechanisms highlight the importance of TAK1 for the activation of NF-κB and MAP kinase family members [38]. The TLR2- and TLR4-associated MyD88-dependent pathway needs an additional adaptor molecule (TIRAP, i.e., MAL) to provoke an inflammatory response [39].

### 2.2. The TRIF-Dependent Pathway

MyD88-deficient macrophages and DCs fail to produce inflammatory cytokines after stimulation of TLR2, TLR5, TLR7, and TLR9 by their respective ligands; this observation points to the dependence of these TLRs upon MyD88 for their activation [40,41,42]. Nonetheless, TLR4 yields a delayed response in MyD88-deficient cells upon LPS stimulation; this observation indicates the existence of an alternative pathway associated with TLR4 [43]. This finding led to the discovery of another adaptor molecule, TRIF, which responds to the TLR activation independently of MyD88 and can activate all three transcription factors i.e., NF-κB, AP-1, and IRFs unlike MyD88 which cannot activate IRFs [44]. TRIF-deficient mice show reduced production of inflammatory cytokines upon LPS stimulation, indicating the positive regulation of MyD88 by TRIF [45]. The interaction between TLR4 and TRIF is mediated exclusively by TRIF-specific adaptor TRAM [46]. TLR3 ligand poly-IC yields a normal response in MyD88-deficient cells but not in TRIF-deficient mice. Of note, TRAM-deficient cells also produce a normal response, which confirms that TRIF is the only adaptor for TLR3 [46]. TRIF contains a Rip homotypic interaction motif (RHIM) at its C terminus to interact with receptor interacting protein (RIP) family members [47]. TLR3-mediated activation of NF-κB and inflammation-responsive genes is abrogated in RIP1-deficient cells, highlighting its involvement in NF-κB activation [47]. Nonetheless, RIP3 disrupts this interaction to inhibit the TLR3 signaling pathway [47]. At its N terminus, TRIF contains three TRAF6-binding domains to interact with TRAF6 with subsequent activation of NF-κB. The mutational studies confirm the participation of both TRIF–RIP1 and TRIF–TRAF6 pathways in the activation of NF-κB via convergence at the IKK complex [48].

## 3. Representative Diseases Associated with TLRs

A wide spectrum of diseases is associated with TLRs, which directly or indirectly aggravate these conditions. Recently, many accomplishments have been made regarding the TLR involvement in several diseases [49,50,51,52]. Here, we will provide a brief overview of the influence of TLRs on autoimmune, inflammatory, and malignant diseases [53,54,55,56].

Sepsis, being the leading cause of death in the United States, is the outcome of worst host–pathogen interactions [57,58]. The hyperactive immune response because of the infection by Gram-negative and Gram-positive bacteria leads to septic shock and multiorgan failure [59]. The possession of TLR2 and TLR4 ligands, especially the TLR4 ligand (LPS), by these bacteria makes a significant contribution to the development of sepsis. On the other hand, the human body’s own immune response but not infection itself is mainly responsible for septic shock [60]. Several TLR inhibitors and new modalities are being developed to control sepsis [61].

Chronic obstructive pulmonary disease (COPD) is described as bronchial inflammation and poor reversible air-flow [56,62]. The interaction of TLRs with attacking viruses can worsen this condition, and patients with higher levels of inflammatory cytokines such as CCL5 and TNF-α have been described in the literature [63]. The inhibition of TLRs is one of the treatments of COPD [50].

Rheumatoid arthritis (RA) is a well-known inflammatory disease associated with TLRs. With yet unclear pathogenesis, this disease is believed to be related to PAMPs of the commensal microflora, which lead to the hyperupregulation of inflammatory cytokines [64]. After an initial encounter with a PAMP, the condition worsens via the autocrine exacerbation by metalloproteinases (MMPs). Moreover, peptidoglycan and DNA from intestinal bacteria also contribute to RA [65]. This action damages the affected cells thereby releasing proteins featuring damage-associated molecular patterns, e.g., HMGB1, S100-A8, and RNAs, which aggravate the condition by further activating TLRs.

Systemic lupus erythematosus (SLE), simply known as lupus, is an autoimmune disease characterized by the presence of autoantibodies against the nucleic-acid-bound proteins and double-stranded DNA; however, the initial mechanism is still unclear [66]. SLE patients cannot get rid of apoptotic cells and the build-up of immune complexes inside their body; these problems cause lupus by activating endosomal TLRs. Nevertheless, TLR9 has been reported to have a regulatory role in TLR7-mediated inflammation in a subgroup of SLE patients [67,68,69].

Sjogren’s syndrome is an autoimmune disease where the fluid-secreting glands, e.g., salivary glands, are destroyed by the body’s own immune system, and this process potentially involves the TLRs. Patients with Sjogren’s syndrome have higher expression of TLRs, which lead to the hyperactivation of inflammatory genes especially by the activation of TLR7 and TLR9 [70,71].

Cancers are complex diseases, and TLRs’ involvement acts as a double-edge sword in these diseases. The activation of TLR(s) can either exaggerate or suppress cancer depending upon the extent of activation, type of cancer, and a cancer microenvironment [49]. Moreover, TLR-mediated inflammation and cancer have a strong correlation during initiation or exacerbation of various diseases [72]. This observation might be the reason why organs such as the gastrointestinal tract and skin, which contain more PAMPs, are at a higher risk to TLR-mediated oncogenesis. In this regard, TLR4 has been demonstrated to enhance colon cancer, whereas TLR4 deficiency lessens the signs of inflammation and tumor load [73,74]. TLR4 activation is also positively linked with liver cancer progression [75]; however, its role is dependent upon the environment in case of skin cancer [76,77]. TLR4 activation by the LPS from *Helicobacter pylori* increased the proliferation rate of human gastric cancer cell lines [78]. In addition to increased cell survival and proliferation, TLR4 activation on breast and cancer cells can release factors (such as MMPs, NO, VEGF, IL-6, and IL-12) and ligands (such as B7-H1 and B7-H2) which are responsible for immune evasion of cancerous cells [79,80,81,82]. Activation of other TLRs, such as TLR2, TLR5, TLR7, TLR8 and TLR9, has been reported to enhance proliferation in various cancers including liver, gastric, lung, and breast cancer [83,84,85,86,87]. Similarly, cellular transformation in the case of breast cancers is also attributed to the involvement of TLRs [88] because they modify the metabolism of a tumor microenvironment [89], which can favor pro- or antitumor signaling networks [90,91,92].

## 4. TLR-Targeting Therapeutics

TLRs are promising targets for drug development because of their involvement in inflammation, pathogen clearance, cancer, and many other related diseases. Therefore, many pharmaceutical companies are developing peptide- (protein-), chemical-, or aptamer-based modulators specific to TLRs. The natural drugs like peptides have more specificity, higher molecular weight (>1 kDa), and longer half-life than small molecule-based drugs. Moreover, they can be modified for better activity by using some non-natural amino acids. Nevertheless, as compared to many small-molecule drugs, biologics are generally less stable and are likely to undergo aggregation [93], oxidation, or deamidation [94]. Small molecules have more stability, lower molecular weight (<700 Da), oral administration, lower price, nonimmunogenicity, and accessibility to intracellular targets [95]. Aptamers are nucleic acid-based structures which have advantages over peptides and small molecules in certain applications like to detect toxin, non-immunogenic targets [96]. The ideal candidates to target TLRs are scaffolds of naturally occurring modulators: this is a productive approach to the clinical development of TLR modulators.

### 4.1. TLR1/2 and TLR2/6

The heterodimeric coexistence of TLR2 with TLR1 and TLR6 enables it to interact with diverse ligands including lipoproteins, glycoproteins, peptidoglycan, and zymosan [97]. Moreover, TLR2 is thought to be functionally ubiquitous because of its expression in immune, epithelial, and endothelial cells [98]. This observation makes TLR2 an attractive therapeutic target in multiple diseases. The compounds being evaluated in clinical trials include antibodies, lipoproteins, and lipopeptides (Table 2). The most recent ligands, such as OPN-305 (antagonistic antibody), ISA-201 (agonistic peptide), and CBLB612 (agonistic lipopeptide), are in phase 2 clinical trials for cancer therapy and are being used as a drug and adjuvant [99,100]. Potential adverse effects of small-molecule drugs can be overcome by replacement with biologics such as TLR2-targeting monoclonal antibodies (OPN-305) [99].

There is cavity formation on the convex side of the binding site of TLR2 for TLR1 or TLR6; this cavity allows for the docking of TLR2 modulators including Pam3CSK4 [101,102]. There are two lipid and ester chains in the structure of Pam3CSK4. The lipid chains are lodged into the hydrophobic cavity generated by TLR1, while ester chains communicate with TLR2 [101,103]. The interplay of hydrophobic interactions and hydrogen bonding also makes the TLR1–TLR2 complex stable [101]. A mutational study (Met338 and Leu360 to Phe in TLR1) explains the necessity of diacyls for the formation of the TLR2–TLR6 complex.

### 4.2. TLR3

The homodimeric TLR3 generates signals for the production of IFNs in a TRIF-dependent manner after TLR3 is activated by viral infections (double-stranded RNA). Poly-ICLC and derivatives are the only known TLR3-specific agonists currently being evaluated in clinical trials [104,105]. Recently, some other small-molecule drugs were reported to be inhibitors or activators of TLR3 [106,107]. Few clinical trials for antibodies targeting TLR3 have been conducted with asthmatic patients [108]. The success of antibody-based prevention of diseases will lay the foundation for the treatment of endosomal-TLR-related diseases. Nonetheless, an antibody cannot treat the asthmatic condition in patients infected by rhinovirus [109]. Various types of cancers can be treated with TLR3 agonists as an adjuvant therapy with other vaccines or drugs (Table 2).

A synthetic TLR3 agonist, poly-ICLC, is a complex of polyinosinic/polycytidylic acid, carboxymethylcellulose, and poly-l-lysine. The mimetics of a natural ligand of TLR3, dsRNA, are promising candidates for activation of associated TLRs. TLR3 activation is initiated by the interaction of its ectodomains with dsRNA, which relocates the C terminus of an ectodomain for further interactions and stability [110,111]. Moreover, TLR3 activation is caused by its interaction with a ligand backbone rather than sidechains (bases); this mechanism allows TLR3 to be activated by multiple combinations of nucleotides [110].

### 4.3. TLR4

The only TLR functioning on the plasma membrane and in endosomes in both a MyD88- and TRIF-dependent manner is TLR4. This observation enables various options for the design of modulators such as those targeting CD14, LBP, MD2, MAL, and/or TRAM. Of all TLRs, only TLR4 contains an MD2-provided ligand-binding pocket rather than a pocked formed by its own ectodomain; this finding highlights the importance of TLR4 as a therapeutic target [112]. Because of the large hydrophobic cavity of MD2 and suitability for the binding of lipid-A derivatives, disruption of an MD2 interaction with a ligand or TLR4 is regarded as one of the therapeutic options [113]. If we consider the interaction of MD2 and lipid-A, a six-acyl-chain-carrying lipid molecule can completely occupy the pocket; this event reorients a sidechain into the binding pocket to dock TLR4 with MD2 properly for the activation of signaling [114]. Nevertheless, a smaller number of acyl chains inhibits the TLR4 activation because of the failure to reorient the side chains properly [112,115].

Well-known TLR4 modulators include lipid VI-A and its derivatives (glucopyranosyl lipid adjuvant (agonist) [116], lipid 4A (antagonist), and monophosphoryl lipid A (weak agonist)) [117] and peptide- or antibody-based therapeutics [113,118]. The TLR4 modulation holds promise for the treatment of various pathologies including immunological diseases, viral infections, cancers, and inflammation (Table 2).

### 4.4. TLR5

Bacterial monomeric flagella are recognized by the ectodomain of TLR5, thereby eliciting an immune response [119]. After enterobacterial invasion, TLR5 activates the MyD88-dependent pathway thus maintaining the intestinal homeostasis. Most of immune cells there, predominantly mucosal DCs, express TLR5 [120,121]. A recombinant flagellin protein is being used for targeting TLR5 in many clinical trials [122,123,124,125]. Moreover, preclinical studies on a small-molecule inhibitor of the TLR5–flagellin interaction are under way [126]. From the therapeutic point of view, most of TLR5 ligands are being used as adjuvants to enhance the efficacy of vaccine candidates rather than being used as drugs (Table 2). TLR5 detects only protein-based ligands; this property allows researchers to design peptide-based activators and/or inhibitors [124,127].

Recently reported crystal structure of flagellin complexed with zebrafish TLR5 provides insights into the mechanism of TLR5 activation [128]. The residues Arg89, Glu114, and Leu93 in flagellin and leucine-rich repeat 9 (LRR9) of TLR5 are critical regions in the structure of the complex for the interaction; these data could be further explored for designing therapeutics [128].

### 4.5. TLR7 and TLR8

TLR7 and TLR8 are located in the endosomal compartment and activate signaling in a MyD88-dependent manner after being stimulated by single-stranded RNA (ssRNA) [129,130]. Most of the TLR7/8 modulators in clinical studies are small-molecule compounds such as resiquimod, imiquimod, or GSK2245035 (Table 2) [131,132,133]. Because of some structural differences, monocytes and plasmacytoid DCs can be directly activated by TLR7; however, monocyte-derived DCs can be directly activated by TLR8. IFN and the associated cytokine-based antiviral response in human peripheral mononuclear cells (PBMCs) is more potently regulated by a TLR7 agonist rather than by a TLR8 agonist [134]. Nevertheless, proinflammatory cytokines such as TNF-α, IL-12, and MIP-1α are more strongly upregulated by a TLR8 agonist rather than by a TLR7 agonist. TLR7 agonists are being evaluated in phase I/II trials to limit the viral load in HBV- and HIV-infected patients [135]. TLR8 can be activated by a synthetic small-molecule ligand (motolimod (VTX-2337)) and a natural ligand (ssRNA), and they are currently assessed in clinical trials [130,136]. VTX-2337 has been tested for the treatment of various cancers such as colorectal cancer, head and neck cancer, melanoma, pancreatic cancer, renal cell carcinoma, breast cancer, and non-small cell lung carcinoma. This compound has been tested alone and in combination therapy for lymphoma [136].

Both TLR7 and TLR8 contain Z-loops to recognize ssRNA and two binding sites: the first one to bind guanosine (G) and uridine (U) in TLR7 and TLR8, respectively, and the second site to bind ssRNA in both [137]. This observation reveals the similarity in their mechanism of action on signaling. The binding of ssRNA in TLR7 primes it to bind guanosine for subsequent dimerization, whereas synthetic molecules, such as R848, do not require ssRNA for the TLR7 activation [138,139]. Moreover, TLR7 exists in a monomeric form in the absence of a ligand and dimerizes after recognizing the respective ligand, whereas TLR8 is a weak dimer without a ligand and undergoes conformational changes after recognizing the ligand to activate downstream signaling. The reason might be the regulatory role of Z-loops in TLR8 because their cleavage from TLR8 can activate signaling in the absence of a ligand via the formation of a tight functional dimer [140].

### 4.6. TLR9

CpG DNA is recognized by endosomal TLR9 to activate the IFN response [141,142]. Because of its involvement in multiple diseases, many approaches have been employed to design relevant therapeutics. All the TLR9-specific ligands in clinical studies are nucleotides and their derivatives. AZD1419, a CpG-based TLR9 agonist, stimulates IFN production for the treatment of asthma and is considered safe for treating various diseases in humans [143]. Similarly, some other TLR9 agonists such as CYT003, EMD 1201081, and GNKG168 have been tested clinically against various cancers (Table 2) [144,145,146].

TLR9 also forms a symmetrical complex with its ligand like other TLRs do; however, it maintains its monomeric form during an inhibitory interaction with an antagonistic ligand. There is a symmetric interaction in the 2:2 stoichiometric ratio between CpG-DNA and TLR9, specifically via the carboxy terminus (LRR20–22) with one protomer and the amino terminus (LRRNT–LRR10) with the other [147]. On the other hand, TLR9 inhibition is mediated by the binding of CpG-DNA to the concave surface (LRR2–10) of TLR9.

### 4.7. TLR10–TLR13

Along with above-mentioned TLRs, humans also possess TLR10 and TLR11; however, they do not have TLR12 and TLR13 [148]. TLR10 expression has been observed in various human cells and organs such as monocytes, neutrophils, B cells, lymph nodes, and the spleen; however, its specific ligand and function are not known yet [149]. Recently, the anti-inflammatory rather than proinflammatory nature of TLR10 was revealed because of modulation of the TLR2 response via heterodimer formation with TLR1 and TLR6 [150]. A pseudogene of TLR11 with a premature stop codon has been found in humans; it is unable to express a functional protein [151]. Murine TLR11 and TLR12 are reported to recognize profilin from *Toxoplasma gondii* by forming a heterodimeric structure [148].

## 5. Controlled Drug Delivery Systems

Drug delivery is the procedure of administering a medicinal product to attain a therapeutic outcome in humans or animals. Controlled-drug release systems got their start in the 1950s with the advancement of transdermal and oral constant-release systems. To achieve this objective, different drug delivery systems have been devised and are being investigated [152]. Polymeric materials are utilized for this purpose and allow for increased circulation time, solubility of otherwise insoluble drug molecules, increased in vivo stability, site-specific targeting, a confined release, easier clearance from kidneys, and reduced adverse effects [153,154,155]. A drug can be delivered via polymers by conjugation, encapsulation, or an embedding method [156,157]. For these systems, the drug delivery rate is controlled by the degradation rate of the polymer or the diffusion rate of the drug through a polymer matrix. Presently applicable polymer-based drug delivery systems can be grouped into five types based on their mechanism: diffusion-controlled systems, solvent-activated systems, chemically controlled systems, magnetically controlled systems, and targeted drug delivery system.

### 5.1. The Diffusion-Controlled System

One of the vital processes in the body and nature is the exchange of materials via diffusional mass transport. This phenomenon was first illustrated by Adolf Eugen Fick (1829–1901) in a quantitative way [12]. Based on the internal network of a drug delivery system and loading of a drug into it, diffusion-controlled systems are classified into two major categories [158]. If the drug is in the center and the drug-releasing material (usually a polymer) is partitioned in consonance with the core shell framework, then this is called a “reservoir system.” In contrast, if the drug is uniformly dispersed in a continuous polymeric matrix, held together by a drug release-regulating material, then this is called a “monolithic system” [158]. Both systems contain noncovalently bonded drugs that are embedded or incorporated inside their polymeric matrices [159] and are further subdivided into two main subtypes. Reservoir devices can be porous or nonporous: In porous devices, the drug has to diffuse through the pores filled with oil or water; however, in nonporous devices, the drug has to pass via diffusion through the membrane of the polymer. Monolithic devices are further classified based on the concentration of the loaded drug: In monolithic solution devices, drug concentration in the matrix is equal to the solubility of the drug if the partition coefficient of the drug is 1.0; in contrast, in monolithic dispersion devices, drug concentration in the matrix is higher than its solubility. In a reservoir system, the integrity of the polymeric membrane is highly important because its accidental rupture may cause a sudden discharge of the drug, a phenomenon known as drug dumping [160]. Moreover, it is necessary to remove the intact polymer of the reservoir from the body after the drug has been depleted. In contrast, the monolithic system ensures a uniform release of the drug without the risk of drug dumping.

### 5.2. The Solvent-Activated System

This system is associated with one of two phenomena: osmosis or swelling. The osmotically driven system depends upon the manufacturing material of the semipermeable membrane through which the solvent flows into the drug-carrying chamber, and the difference in osmotic pressure between the two sides of the membrane. An external fluid has a lower drug concentration and moves inside the device (having a higher drug concentration) through a membrane. The drug inside the system diffuses outside through an orifice with velocity that depends on the amount of water being absorbed by the polymeric matrix [161]. The swelling system involves a hydrophilic polymer that forms a three-dimensionally organized structure after absorbing water without dissolving in it. There are three driving forces in this process: a polymer stress gradient, a water concentration gradient, and osmotic forces. These systems allow for a zero order and environment-independent release of the drug without reformulation of the material for different drugs. Nevertheless, these can be more expensive and require more quality control.

### 5.3. The Chemically Controlled System

This system involves a polymer–drug conjugate in which drug molecules are attached through spacer molecules to the polymer backbone. Inside the body, the bond between drug and polymer carrier is broken by either enzymatic cleavage or other hydrolysis. Diverse types of hydrolysable and biodegradable chemical linkages are used to affix the desired drug to the polymer backbone [162]. Usually these polymer–drug conjugates have a transport system to direct the polymer to target tissues or organs. Aside from cleavable and hydrolysable polymers, chemically controlled systems also use biodegradable or bioerodible polymers. The difference between the two systems depends upon the mechanism of polymer degradation. The biodegradation involves the cleavage of polymer chains with a subsequent decrease in size; however, bioerosion is mediated by the bulk or surface erosion of the polymer with a subsequent decrease in size. In all these cases, the drug release depends on the composition of the polymer, so the polymer can control the kinetics of the drug release into a target area [163].

### 5.4. The Magnetically Controlled System

This system involves fabrication of the drug-carrying polymeric matrix with a tiny magnetic ring. The outer surface is covered with a drug-impermeable polymer except for one central cavity. After application of an external magnetic field, the magnet starts vibrating and releasing the drug molecules at the desired rate. On the other hand, to gain efficient control over the particle motion, it is necessary to overcome the hemodynamic force by the external magnetic force. Thus, weak magnetic fields should be applied in case of an in vivo application. Metals such as iron, nickel, and cobalt are commonly used for this purpose [164].

### 5.5. A Targeted Drug Delivery System

Despite the effectiveness of the above-mentioned methods, they are not applicable to all kinds of drugs and associated diseases. There is a need to optimize other delivery systems including self-regulated insulin delivery vehicles, protein delivery systems, poorly soluble formulations, and targeted delivery systems. Among these, targeted drug delivery systems have received a lot of attention because they involve nanotechnology-based delivery systems. Two types of approaches are mostly used for targeted drug delivery: active targeted drug delivery and passive targeted drug delivery.

#### 5.5.1. Active Targeted Drug Delivery

In this system, delivery vehicles (such as nanoparticles) are more specific to the target area. This is achieved by getting the information about the receptors of the target cells and by attaching the receptor-specific ligand to the vehicle. For example, tumor cells are targeted by conjugating transferrin to the nanoparticles that mediate transferrin–receptor endocytosis after interaction. This conjugation process increases drug delivery efficiency as compared to the nonconjugation method. Magneto liposomes are another method of targeted drug delivery and act as a contrast agent in magnetic resonance imaging. Liposomes conjugated to a desired drug can be delivered to the target area through the mechanism of magnetic positioning [165]. Moreover, activation of targeted nanoparticles can be implemented by target-specific triggers such as pH and redox potential. Some special areas or cellular compartments in the body have pH different from that of most of the other body parts. This difference can be exploited by trigger-based nanoparticles to release a loaded drug in only specific areas. Tumor cells create hypoxic conditions by altering the redox potential in the surrounding area. Redox-sensitive nanoparticles are utilized to selectively release the drug in the tumor area [166].

#### 5.5.2. Passive Targeted Drug Delivery

In this process, the efficacy of a drug is directly linked to circulation time [167]. To reach this goal, nanoparticles are covered by a distinct type of covering such as polyethylene glycol (PEG). This approach allows for the linkage of water molecules to the oxygen atom on the PEG surface through hydrogen bonding, which imparts hydrophilic properties to the surface. Because of the establishment of a thin water film on the surface of nanoparticles, they are protected from phagocytosis. Moreover, drug-carrying molecules remain longer in the circulation because of hydrophobic interactions common in the reticuloendothelial system [165]. Nanoparticles having a size of 10 and 100 nanometers are recommended for the longer persistence in the systemic circulation [166].

Via application of both active and passive targeting, drug-carrying nanoparticles can have major advantages over a conventional drug. The circulation time of nanoparticles inside the body is prolonged substantially unless they are strongly drawn to their target via magnetic positioning, cell-specific ligands, or pH-responsive materials. Because of specific targeting by nanoparticles, there are fewer adverse effects [163].

### 5.6. Examples of Targeted Drug Delivery

The efficacy of a designed drug mainly depends upon its half-life, specificity, and delivery to a target site. Half-life and specificity are the characteristics of the drug itself; however, targeted delivery depends on the target location. It is relatively easy to target the cell surface rather than intracellular compartments. Many approaches have been developed for efficient drug delivery with minimal off-target effects; some of which are discussed above in detail.

TLRs are mainly expressed on immune cells such as monocytes, macrophages, and DCs. The overactivation of TLRs in these cells can cause inflammation, which is a cause of various diseases such as asthma, COPD, cancer, tuberculosis, and HIV infection. The on-target delivery of drugs to activate or inhibit the key factors is the ultimate goal in these cases and may result in a cure. It is more challenging to target intracellular compartments because of the outer barrier in the form of the plasma membrane, especially in the case of gene therapy.

Liposomes are considered one of the delivery systems for phagocyte-associated therapies to deliver drugs into target cells. Liposomes have the advantage of biocompatibility, low immunogenicity, good drug protection, and cell specificity. Nonetheless, they also possess some limitations such as short shelf life, high cost, poor scale-up, and in some cases off-target effects and toxicity. They should be protected from macrophages by shielding with PEG if the target is other than macrophages [168]. The following physicochemical properties of liposomes contribute to the successful delivery of drug molecules into cells. (1) Size: some studies show greater uptake of small liposomes (<100 nm) [169] while others illustrate a direct relation of size with the ease of uptake [170,171]. (2) Charge: cationic liposomes containing stearylamine induce apoptosis in RAW 264.7 macrophages via the mitochondrial pathway by stimulating the production of reactive oxygen species, cytochrome C release, expression of caspases, and activation of protein kinase C (PKC) [172,173,174,175]. These findings have shifted the trend toward anionic and neutral liposomes. Macrophages preferentially recognize negatively charged liposomes such as phosphatidylglycerol and phosphatidylserine [169]. A comparison of liposomes containing phosphatidylserine (anionic) and phosphatidylcholine (neutral) has revealed enhanced internalization of negatively charged liposomes by macrophages [176]. (3) pH: Liposomes with pH-sensitive properties are used to carry plasmid DNA to RAW 264.7 cells [177]. Recently developed amphoteric liposomes, Nov038, are employed to deliver antisense oligonucleotides to an inflammatory site in experimental arthritis. They are anionic at neutral pH and cationic at low pH thereby avoiding nonspecific interactions in blood and facilitating complexation with nucleic acids, respectively [178]. (4) Ligands: The specificity and uptake of liposomes can be improved by the addition of some ligands such as peptides, antibodies, proteins, polysaccharides, or glycoproteins. Cell-penetrating peptides and cell-targeting peptides have been linked to liposomes to enhance cellular uptake and specificity, respectively, for various cell types [179]. Conjugation of GGP-peptide (GGPNLTGRW) to liposomes enhances its specificity to monocytes and neutrophils [180,181]. Similarly, integrin receptors on monocytes can be targeted more specifically by the addition of RGD-peptide (Arg–Gly–Asp) to liposomes [182,183]. Conjugation of nonspecific or monoclonal antibodies to liposomes (immunoliposomes) opsonizes the liposome, and this event may activate the complement system with subsequent enhanced uptake by phagocytes [184,185]. The conjugation of IgG and IgM to liposomes (nonimmunoliposomes) can cause their opsonization in vivo for enhanced uptake by macrophages [185]. Mannosylated liposomes can target immune cells by interacting with the C-type lectins expressed by them; this approach enhances the in vitro and in vivo uptake of liposomes [186]. Mannosylated liposomes have been used to deliver anticancer agents, a nuclear factor-B (NFB) decoy, and an anti-inflammatory drug, dexamethasone palmitate [186,187].

Nanoparticles have been employed to deliver immunotherapeutic drugs to target cells. An immune-cell response can strongly inhibit the progression of cancer; therefore, targeting these cells holds great promise for cancer treatment. In this regard, TLR agonists are used as adjuvants to treat various cancers [188]. Aside from their role in an innate immune response, TLRs can trigger adaptive immunity by activating CD8^+^ T cells and CD4^+^ T helper (T_H_) cells by priming antigen-presenting cells [189]. Because of the TLR3-, TLR7-, and TLR9-mediated CD8^+^ T-cell response in T_H_1 mode, agonists specific to these TLRs are used for cancer nonvaccines [190,191]. A TLR9 agonist, CpG, has been tested for enhanced immune activation via conjugation with a nanocarrier or cationic polymer [192,193]. The cationic antigen peptide and anionic TLR3 agonist poly-I:C have been coloaded onto gold nanoparticles for a robust antigen-specific response of CD8^+^ T cells in vivo [194]. The synergy among various TLR agonists has been achieved via multifaceted drug loading by means of relevant nanoparticles [195]. Moreover, TLR agonists and small interfering RNAs have been codelivered using nanoparticles against cancer [196,197].

Hydrogels are three-dimensional hydrophilic polymers capable of absorbing a large amount of physiological fluid while maintaining their networking structure [198,199]. Instead of macroscopic hydrogels, there is a huge interest in microscopic and nanoscopic hydrogels which are known as microgels and nanogels, respectively [200,201]. Phase I clinical trials of amphiphilic cholesterol-modified pullulan nanogels carrying a truncated HER2 protein have been conducted as cancer vaccination via a T-cell immune response and antibody response against HER2 [202,203]. The toxicity of the intracellularly targeted drugs can be minimized by means of the degradable nanogels, which are affected by various stimuli such as pH, enzymatic activity, or reducing agents [204]. The acrylamide nanogels copolymerized with an acid cleavable bisacrylamide acetal crosslinker can be used to deliver TLR9-targeting CpG DNA into antigen-presenting cells [205,206].

## 6. Drug Delivery Vehicles

**Figure 2:**
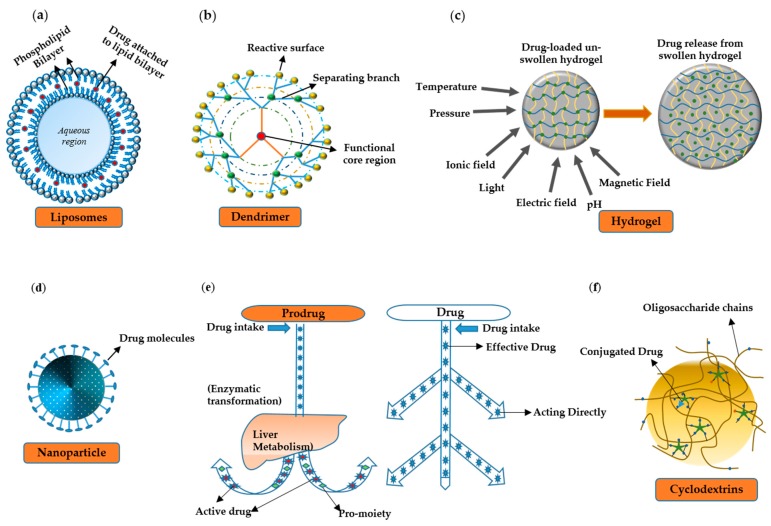
Drug delivery vehicles. (**a**) Liposomes are composed of phospholipids or other small-molecule compounds with various sizes, composition, and drug-loading capability. A lipid-soluble drug is attached to the lipid bilayer for safe and reliable transport to a target tissue. (**b**) Dendrimers are abundantly branched macromolecular structures having three major components: the core, branches, and a reactive surface, which has drug-entrapping properties. (**c**) A hydrogel is a three-dimensional structure consisting of a hydrophilic network of polymer chains, where drug molecules are attached to the polymeric chains. After exposure to the specific stimuli in an aqueous environment, the hydrogel swells and releases drug molecules into the surrounding environment. (**d**) Nanoparticles are small particles ranging in size from 1 to 100 nm where drugs are attached to the surface. (**e**) A prodrug is an inactive form of a drug and is activated after enzymatic or chemical metabolism inside the human body by releasing a promoiety. This property makes the drug a suitable candidate for specific targeting and controlled release. (**f**) Cyclodextrin represents a family of cyclic oligosaccharides with a hydrophilic outer surface and a lipophilic central cavity.

Vehicles are primarily used for the targeted delivery of drugs. The targeted approaches have three main advantages over conventional ones: (1) more stability and solubility; (2) better pharmacokinetic properties due to longer half-life, better absorption, and low volume for distribution; (3) better pharmacodynamic properties due to more specificity and high therapeutic index. An ideal drug delivery vehicle should possess qualities such as biocompatibility, nontoxicity, biodegradability, nonimmunogenicity [207], and resistance to phagocytosis by the host’s defense system [208]. It must cross blood–brain barrier and tumor vasculature (for tumor chemotherapy). It must be selectively and specifically recognized by the target cells while maintaining the specificity of other surface ligands. The ligand–drug complex must keep stability in interstitial fluid, plasma, and other body fluids. The vehicle must release the drug inside target cells, tissues, or organs [209,210]. There are different types of drug delivery vehicles that carry the drug to a diseased tissue. A few of them are discussed below (Figure 2).

### 6.1. Liposomes

Liposomes are composed of either natural or synthetic phospholipids. The prevailing chemical and physical attributes of a liposome are confined to the characteristics of the constituent phospholipids including charge density, permeability, and steric hindrance [211]. Drug loading within liposomes can be accomplished by (i) the use of organic solvents and solvent exchange mechanisms; (ii) liposome formation in an aqueous solution saturated with a soluble drug; (iii) pH gradient methods; and (iv) the use of lipophilic drugs [212].

Generally, liposomes reach their site of action from the bloodstream by exiting into the interstitial space. They achieve specific targeting by both active and passive targeting approaches. This is due to their smaller size (~400 nm) and additional outer covering with other molecules such as PEG. PEGylation increases the circulation half-life of liposomes by lowering clearance by the mononuclear phagocyte system. The delivery mechanism by liposomes involves their fusion to the plasma membrane with a subsequent release of a drug inside the cell [213]. Nevertheless, liposomes face the problems of instability, poor skin permeation, sterilization, and difficulties with large-scale production [214,215,216,217].

Proliposomes were introduced in 1986 to improve the stability of conventional liposomes [218]. They are a smart substitute of conventional liposomes and consist of dry and free-floating particles that form a liposomal suspension on contact with water molecules. The solid nature solves the stability problem of conventional liposomes without affecting their intrinsic characteristics. They consist of three main components: a phospholipid, drug, and porous powder whose size controls the size of reconstituted liposomes [219,220,221]. They can be synthesized by many methods such as film deposition on carriers [222], crystal–film [223], powder bed grinding [224], fluidized-bed [225], spray drying [226], and freezing and drying [227].

Niosomes (another version of liposomes) are spherical vesicles primarily consisting of nonionic hydrated surfactants, most commonly cholesterol (CHOL) and its derivatives. The exclusive architecture of niosomes makes them well suited for encapsulating both lipophilic and hydrophilic drugs. This can be done via absorption of the hydrophilic part in an aqueous core; meanwhile, the lipophilic material is sheathed by subdivision into a lipophilic sphere of the niosome bilayer [228]. Generally, niosomes are synthesized by means of convenient and accessible surfactant materials [229]. Hydrophilic–lipophilic balance is a dimensionless parameter that describes drug-entrapping capability and helps to regulate this property [230]. Besides the surfactant composition, the methods of niosome preparation and drug encapsulation are additional criteria for the self-assembly of surfactants into niosomes [231]. Depending upon the size of niosomes, they can be classified into three basic types. Unilamellar small vesicles range in size from 10 to 100 nm; unilamellar large vesicles range in size from 100 to 3000 nm; and multilamellar vesicles are composed of more than one bilayer [232]. A variety of methods are being used to form niosomes: the thin-film hydration method [233], hand-shaking method [234], the “bubble” method [234], ether injection method [234], reverse phase evaporation method [235], sonication method [236], microfluidization method [234], heating method [237], freeze and thaw method [235], dehydration rehydration method [231], and proniosome technology [238]. The drug-loading mechanism for niosomes includes a direct entrapment (passive loading) method and a remote loading (active loading) method; the former is the simpler one, whereas the latter boosts the efficiency of drug loading with the help of ions and pH [239]. The drug release mechanism of niosomes depends on the concentration, course of administration, presence time, and effects of the drug in organs such as the liver, spleen, lungs, and bone marrow. Niosomal modification with polyethylene glycol (PEG) causes them to last for a longer period in circulation [239]. Niosomes were first used in the cosmetics industry but now have caught the attention of pharmaceutical companies because of their many benefits for controlled drug delivery systems (nonimmunogenicity, biodegradability, and bioavailability), thus being a powerful candidate with excellent properties of the drug release mechanism [240] and encapsulating a variety of drugs, e.g., insulin, DNA vaccine, doxorubicin, hemagglutinin, EGFP, ovalbumin, α-interferon, etc. [233].

### 6.2. Hydrogels

A hydrogel is a cross-linked polymeric network material produced by a reaction of one or more monomers and can swell and preserve a large quantity of water without being dissolved. Hydrogels have gained huge recognition in the last 50 years owing to their extraordinary role in drug delivery and a wide range of other applications [241]. Hydrogels can be produced from synthetic as well as natural polymers. Synthetic polymers have hydrophobic properties and are much stronger than natural polymers. Their stability and deliberate degeneration are implemented by the mechanical durability of the material which in turn depends on its optical design [242]. The water-absorbing property of hydrogels is due to the hydrophilic functional groups attached to the polymeric backbone, whereas their insolubility is a consequence of cross-links among network chains [243]. Almost all hydrogels are glassy when dehydrated, and drug discharge mostly occurs after simultaneous water and drug retention through a swelling-based controlled-release system [244]. Different authors have recommended a novel class of hydrogels that feature both a temperature- and pH-sensitive swelling mechanism. These materials can yield tremendous results in protein drug delivery and enzymatic processes [245]. N-alkyl acrylamide is a very common class of monomers that is being used in the production of temperature-sensitive hydrogels. This monomer has various side chains which have a useful link with water via hydrogen bonding [246].

### 6.3. Prodrugs

Prodrugs are chemical byproducts that require one or two enzymatic or chemical conversional steps to form an active drug. In some situations, a prodrug is composed of a single compound in which two pharmacologically active drugs are joined together and this single molecule serves as a promoiety for other derivatives such as codrugs [247]. Prodrugs give various opportunities to scientists to overcome numerous hurdles on the path of drug production and release, e.g., chemical instability, low aqueous solubility, insufficient oral absorption, poor brain penetration, local irritation and toxicity, and fast presystemic metabolism. The prodrug format can also enhance drug targeting and life cycle management after improvement of the drug release properties of existing prodrugs [248]. The most inspiring feature of a prodrug is that it is site selective. This site selectivity can be accomplished in four ways: (i) selective metabolic activation through enzymes, (ii) transporter-mediated delivery, (iii) antigen targeting, and (iv) passive drug enrichment in an organ [249].

### 6.4. The Nanoparticle System

Nanocapsules are vesicles in which a drug is uniformly and physically diffused inside a cavity ringed by a polymeric membrane. Nanoparticles range in size from 10 to 1000 nm. To produce nanospheres or nanocapsules, the drug is either entrapped, dissolved, encapsulated, or affixed to a nanoparticle matrix [250]. Nanoparticles can be prepared by various methods including polymerization of poly(alkyl cyanoacrylate) (despite being biodegradable, it is well tolerated in vivo) [251], the solvent extraction method (good for a laboratory scale operation) [252], salting out emulsification method, and supercritical fluid technology (this method is advantageous because the solvent-free solute is precipitated) [253]. The main purpose of an active nanoparticle delivery system is to lower the drug dose required to accomplish a distinct therapeutic outcome and thereby to reduce the cost and reduce the adverse effects. The two complementary and synergistic attributes of organic and inorganic nanostructured materials are extensively used in a drug delivery system. On one hand, hard nanoparticles constructed of an inorganic material (such as mesoporous and gold particles and quantum dots) can be used for the detection and diagnosis of a pathology inside diseased tissues. On the other hand, soft nanoparticles constructed of an organic material (such as liposomes and amphiphilic polymers) offer improved characteristics to deal with physicochemical conditions in pathological and healthy tissues [254]. Appreciable developments in biodegradable nanoparticles have transpired in the past few decades. To enhance the therapeutic efficacy, numerous polymers have been tested in drug delivery research to effectively carry a drug to a target site with fewer adverse effects [255].

### 6.5. Dendrimers

These are hyperbranched three-dimensional nanosized molecules composed of branching groups covalently attached to a pivotal core, arranged in monocentric layers that end with various externally activated functional groups [256]. In solution, the structure of dendrimers can be determined by various factors such as the spacer length, generation, surface modification, ionic strength, temperature, and pH [257]. They can boost the bioavailability and solubility of hydrophobic drugs that are conjugated to their surface functional groups or entrapped in their intramolecular cavity. Their surface modification with peptides, sugar groups, monoclonal antibodies, or folic acid can be employed for site-specific delivery of a drug [258]. Analysis of the association between inclusion components and dendrimers is an essential step for the advancement of this novel technology [259]. Dendrimers are useful as a carrier for gene therapy and move into the cell through endocytosis with subsequent degradation by lysosomes [260]. The targeted cargo genes are then discharged and penetrate the nucleus to perform their work in gene therapy [258].

### 6.6. Cyclodextrins

Cyclodextrins are a group of cyclic oligosaccharides and have tremendous applications in the pharmaceutical industry. They are synthesized from glucose-containing compounds, e.g., d-glucopyranoside building blocks that have both α-1,6-glycosidic and α-1,4-linkages. Cyclodextrins have three-dimensional structure, which makes them suitable for pharmaceutical applications. The enormous number of hydroxyl groups makes cyclodextrins water soluble, while their hydrophobic cavity allows for encapsulation of various lipophilic molecules ranging from ions to small-molecule drugs, oligonucleotides, and proteins [261]. At a laboratory scale, cyclodextrins enhance drug delivery through membranes if specific parameters are considered, e.g., unstirred water layer [262].

## 7. Concluding Remarks

TLRs are involved in various autoimmune, inflammatory, and malignant diseases. It is worthwhile to design drugs targeting TLRs and their associated members participating in TLR signaling pathways. The designed drugs must be safe, highly specific, and effective to treat the patient. Few such drugs are mentioned above; however, there is always room for improvement of the existing ones via deep analysis of the structure of a target molecule and via improvement of drug discovery approaches. Even after successful in vitro evaluation, sometimes the drugs do not work as expected in vivo. They might also show adverse effects because of their nonspecific unloading inside the human body. Therefore, selection of an appropriate delivery method is very important for maximal and safe therapeutic benefits. Liposomes, hydrogels, nanoparticles, and other drug delivery modalities must be kept in mind during in vivo evaluation.

## Figures and Tables

**Table 1:** Expression, localization, and ligands of Toll-like receptors (TLRs).

Receptor	Microbial Ligand(s)	Microbial Ligand Source	Endogenous Ligand(s)	Adaptor	TLR Localization	Expression
TLR1/2/6	Triacyl and diacyl lipopeptides, glycolipids, zymosan, lipoteichoic acid	Bacterial lipoprotein and peptidoglycan, fungi, mycoplasma, gram-positive bacteria	HMGB1, HSPs, HDLs, hyaluronan,	MyD88-MAL	Cell surface	DCs, Mon, Mac, B
TLR3	Double-stranded RNA (dsRNA)	Viruses	Self dsRNA	TRIF-TRAM	Endosomes	DCs, L, Plt, B
TLR4	Lipopolysaccharide	Gram-negative bacteria	HSPs, fibrinogen, HA, heparin sulfate, HMGB1, LDLs	MyD88-MAL/TRIF-TRAM	Cell surface	Mon, Mac, N, DCs, M, B, C
TLR5	Flagellin, profilin	Bacteria, *Toxoplasma gondii*	HMGB1	MyD88-MAL	Cell surface	DCs, Mon, Mac, C, IE
TLR7	Single-stranded RNA (ssRNA)	Viruses	Self ssRNA	MyD88-MAL	Endosomes	DCs, Mon, Mc, Plt, B
TLR8	ssRNA	Viruses	Self ssRNA	MyD88-MAL	Endosomes	DCs, Mon, Mac, M, IECs
TLR9	DNA	Bacteria, viruses	Self DNA	MyD88-MAL	Endosomes	DCs, Mon, Mac, Plt, B
TLR10	Triacylated lipopeptides	NA	NA	MyD88-MAL	Cell surface	Mon, N, B, LN, S

Abbreviations: HMGB1, high mobility group box 1; HSPs, heat shock proteins; HDLs, high-density lipoproteins; LDLs, low density lipoproteins; MyD88, myeloid differentiation primary response protein 88; MAL, MyD88-adaptor-like; DCs, dendritic cells; Mon, monocytes; Mac, macrophages; B, B-cell; Plt, platelets; N, neutrophils; M, mast cells; C, cancer cells; IE, intestinal epithelium; IECs, intestinal epithelial cells; LN, lymph node; S, spleen; HA, hyaluronic acid; NA, not available.

**Table 2:** TLR-targeting drugs in clinical trials.

TLR	Ligand	Type	Disease	Mechanism	Phase
TLR2 (with TLR1 or -6)	OPN-305 and derivatives	Monoclonal antibody	Inflammatory disease, myelodysplastic syndrome, kidney transplant rejection, pancreatic tumor	Anti-inflammatory	Phase II
	CBLB6I2	Synthetic lipopeptide	Cancers (breast)	Blood cell recovery	Phase II
	ISA-20I	Peptide	Head and neck tumor	Maturation of DCs	Phase II
TLR3	Poly-ICLC	Synthetic dsRNA	Various cancers (e.g., colon, ovarian, breast, prostate)	Immune stimulation and modulation of tumor microenvironment	Phase I and II
	PRV-300	Antibody	Asthma	Anti-inflammatory	Phase I and II
TLR4	NI-0I0I	Antibody	Rheumatoid arthritis	Anti-inflammatory	Phase II
	GLA and derivatives	Glycolipid	Melanoma, sarcoma, viral infection	Immune stimulator	Phase I and II
	LPS	Glycolipid	Asthma	Inflammation	Phase I
	GSKI79509I	Glycolipid	Cancer	Immune stimulator	Phase I
	Eritoran	Glycolipid	Insulin sensitivity	Anti-inflammatory	Phase II
	CX-0I	Polysaccharide	Leukemia	Microenvironment modulator	Phase I
	PEPA-I0	Small molecule	Cancer	Immune stimulator	Phase II
	PET-lipid A	Glycolipid	Cancers	Immune stimulator	Phase I
	JKB and derivatives	Small molecule	Hepatitis	Anti-inflammatory	Phase II
	MN-I66	Small molecule	Brain injury, glioblastoma	Anti-inflammatory	Phase II
TLR5	Entolimod	Recombinant protein	Cancers	Immune stimulator	Phase I
	Mobilan	Recombinant protein	Cancers (prostate)	Immune stimulator	Phase I and II
	VAX and derivatives	Recombinant protein	Influenza	Immune stimulator	Phase I and II
TLR7	Imiquimod	Small molecule	Various cancers, actinic keratosis, and viral infections	Immune stimulator	Phase I to Phase IV, approved
	GSK2245035	Small molecule	Asthma and rhinitis	IFN production	Phase II
	GS-9620	Small molecule	Hepatitis B	pDC activator	Phase II
	RO702053I	Small molecule	Hepatitis B	Immune stimulator	Phase II
	GSK2245035	Small molecule	Asthma	IFN-α production and immune stimulation	Phase II
TLR8	VTX-2337	Small molecule	Various cancers	Immune stimulator	Phase I and II
TLR9	SD-I0I	CpG-C class oligonucleotide	Lymphoma	Antitumor immune response	Phase I and II
	CYT003	Oligonucleotide	Asthma	TH-I–mediated immune response	Phase II
	MGNI703	DNA-based molecule	HIV and melanoma	Antiviral and antitumor response	Phase I and II
	CpG-7909	Oligonucleotide	Lymphoma, malaria, HIV	Immune stimulator	Phase I and II
	CpG-I0I04	Oligonucleotide	Hookworm infection	Immune stimulator	Phase I
	CpG-ODN	Nucleotide-based	Lung tumor	Immune stimulator	Phase I
	Hydroxychloroquine	Small molecule	Sjogren’s syndrome	Immune suppressor	Phase III
	AZDI4I9	CpG oligonucleotide	Asthma	IFN production	Phase II

Abbreviations: TLR, Toll-like receptor; DCs, dendritic cells; dsRNA, double-stranded RNA; GLA, glucopyranosyl lipid A; IFN, interferon; HIV, human immunodeficiency virus; ODN, oligodeoxynucleotide.
